# Myocardial Fibrosis and Steatosis in Patients with Aortic Stenosis: Roles of Myostatin and Ceramides

**DOI:** 10.3390/ijms242115508

**Published:** 2023-10-24

**Authors:** Elena Zoico, Anna Giani, Tanaz Saatchi, Vanni Rizzatti, Gloria Mazzali, Francesco Fantin, Giovanni Benfari, Francesco Onorati, Silvia Urbani, Mauro Zamboni

**Affiliations:** 1 Division of Geriatric Medicine, Department of Medicine, University of Verona, 37126 Verona, Italy; anna.giani@univr.it (A.G.);; 2 Division of Cardiology, Department of Medicine, University of Verona, 37126 Verona, Italy; 3 Division of Cardiac Surgery, Department of Surgery, Dentistry, Pediatric and Gynecology, University of Verona, 37126 Verona, Italy; 4 Division of Geriatric Medicine, Department of Surgery, Dentistry, Pediatric and Gynecology, University of Verona, 37126 Verona, Italy

**Keywords:** aortic stenosis, ventricular dysfunction, fibrosis, myostatin, ceramides

## Abstract

Aortic stenosis (AS) involves progressive valve obstruction and a remodeling response of the left ventriculum (LV) with systolic and diastolic dysfunction. The roles of interstitial fibrosis and myocardial steatosis in LV dysfunction in AS have not been completely characterized. We enrolled 31 patients (19 women and 12 men) with severe AS undergoing elective aortic valve replacement. The subjects were clinically evaluated, and transthoracic echocardiography was performed pre-surgery. LV septal biopsies were obtained to assess fibrosis and apoptosis and fat deposition in myocytes (perilipin 5 (PLIN5)), or in the form of adipocytes within the heart (perilipin 1 (PLIN1)), the presence of ceramides and myostatin were assessed via immunohistochemistry. After BMI adjustment, we found a positive association between fibrosis and apoptotic cardiomyocytes, as well as fibrosis and the area covered by PLIN5. Apoptosis and PLIN5 were also significantly interrelated. LV fibrosis increased with a higher medium gradient (MG) and peak gradient (PG). Ceramides and myostatin levels were higher in patients within the higher MG and PG tertiles. In the linear regression analysis, increased fibrosis correlated with increased apoptosis and myostatin, independent from confounding factors. After adjustment for age and BMI, we found a positive relationship between PLIN5 and E/A and a negative correlation between septal S’, global longitudinal strain (GLS), and fibrosis. Myostatin was inversely correlated with GLS and ejection fraction. Fibrosis and myocardial steatosis altogether contribute to ventricular dysfunction in severe AS. The association of myostatin and fibrosis with systolic dysfunction, as well as between myocardial steatosis and diastolic dysfunction, highlights potential therapeutic targets.

## 1. Introduction

Aortic stenosis (AS) is one of the most common valvular diseases in the Western world, with an estimated prevalence as high as 12.4% in the elderly [1]. It is known to have significant implications, including reduced quality of life, increased mortality rates, and hospitalization. AS is characterized not only by progressive obstruction of the aortic valve but also by a left ventricular (LV) remodeling response [2,3], leading to impaired relaxation, diastolic dysfunction, and, finally, LV systolic insufficiency [1,2].

Myocardial fibrosis is, in general, one of the hallmarks of a failing heart [4]. Among subjects with severe AS, myocardial fibrosis initially develops as a response to pressure overload, exhibiting an interstitial and reversible nature. However, with the persistence of hemodynamic stress, it becomes an irreversible and substitutive process [4,5,6]. In fact, it is well known that AS does not only affect the aortic valve but also impacts the myocardium, thereby elevating the risk of heart failure [4,6]. Finally, fibrosis may also play a role in the failure of valve replacement surgery [7,8,9].

The activation of profibrotic pathways, which involves a protein of the transforming growth factor-β (TGFβ) family called myostatin, is due to cardiomyocyte death with the consequent release of free fatty acids (FFAs), the recruitment of macrophages, apoptosis, and fibrosis [10]. In LV dysfunction, myostatin results overexpressed [10], and an increased myostatin/IGF-1 ratio has been found in subjects with ventricular dysfunction [11] as well as in adult patients with advanced heart failure across different heart diseases [12,13].

Global longitudinal strain (GLS), assessed with echocardiography, has been considered a surrogate marker for myocardial fibrosis [6]. Studies conducted in a hypertensive rodent model [14] and in a group of patients with AS and control subjects [15] have demonstrated a correlation between GLS and myocardial fibrosis. GLS has been shown to be also an independent predictor of adverse events in patients with severe AS, regardless of preserved or impaired LV systolic function [16]. Interestingly, a reduction in GLS has also been associated with increased epicardial adipose tissue (EAT), which is independently related to increased myocardial fat accumulation and interstitial myocardial fibrosis [16].

Ectopic fat deposition in the myocardium, termed myocardial steatosis, is frequently associated with diastolic dysfunction in severe AS, ischemic heart disease, and various metabolic diseases [17,18]. Generally, excess FFAs are either stored in the adipocytes as lipid droplets (LDs) [19] or converted into ceramides [20,21]. Alterations in perilipins, the main structural constituents of LDs, as well as an increased presence of ceramides, might be linked to heart lipotoxicity and cause cell death and organ dysfunction [22,23,24].

Regarding the role of myocardial steatosis in myocardial dysfunction, animal models have proved that myocardial triglyceride accumulation disrupts normal myocardial lipid metabolism and results in diastolic dysfunction [25]. The role of myocardial steatosis in the development of diastolic dysfunction has also been recently shown in women with coronary microvascular dysfunction and no obstructive coronary artery disease [26]. Pronounced myocardial steatosis has been also observed in severe AS patients, regardless of symptoms, and is independently associated with the extent of impairment in LV strain impairment [17]. In vitro studies have revealed that changes in ceramide synthesis are linked to hypoxia and inflammation. Accumulation of cardiac ceramides occurs in the failing myocardium, and increased levels are also detectable in the circulation of these patients [27]. Conversely, the inhibition of de novo ceramide synthesis has been shown to reduce cardiac remodeling [21].

As shown thus far, both interstitial fibrosis and myocardial steatosis appear to significantly contribute to the development of myocardial dysfunction in different heart diseases and also in patients with advanced AS. However, in human studies, myocardial quality in terms of fibrosis and myosteatosis has been characterized mainly using MRI and histologically only in a few studies to validate MRI measurements [1,6,15].

Thus, the present study aimed to analyze the LV biopsies of patients with severe AS who had undergone surgery, fibrosis, and myocardial lipid infiltration, as well as to investigate the correlation between them and key echocardiographic parameters indicative of LV dysfunction. Furthermore, this study sought to evaluate the underlying mechanisms and pathways correlated with these histological phenomena.

## 2. Results

A total of 31 patients (12 male and 19 female subjects) diagnosed with severe AS undergoing elective cardiac surgery for aortic valve replacement were recruited as our study population. The clinical characteristics as well as the metabolic profiles and echocardiographic data of these subjects are shown in Table 1.

**Figure 1:**
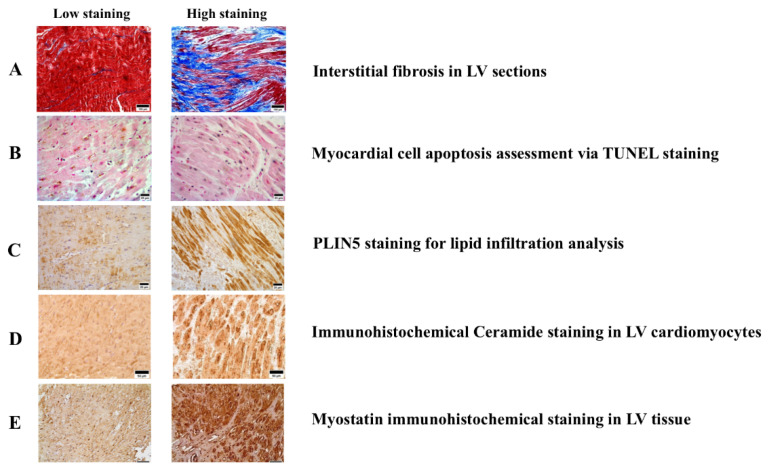
Histological and immunohistochemical analysis of LV tissue sections. (**A**): Interstitial fibrosis in LV sections stained with Masson’s trichrome, where fibrous collagen is shown in blue and myocytes in red (scale bar = 100 μm). (**B**): Myocardial cell apoptosis in LV sections assessed with TUNEL, which highlights nuclei of apoptotic cells in blue or black, and nuclei of non-apoptotic cells in red (scale bar = 25 μm). (**C**): PLIN5 immunohistochemical staining, indicative of ventricular lipid infiltration, with PLIN5 positive cardiomyocytes in brown (scale bar = 25 μm). (**D**): Immunohistochemical ceramide staining of LV cardiomyocytes (scale bar = 50 μm). (**E**): Immunohistochemical staining for myostatin with myostatin highlighted in brown and the nuclei in blue (scale bar = 100 μm). LV = left ventriculum. PLIN = perilipin.

**Figure 2:**
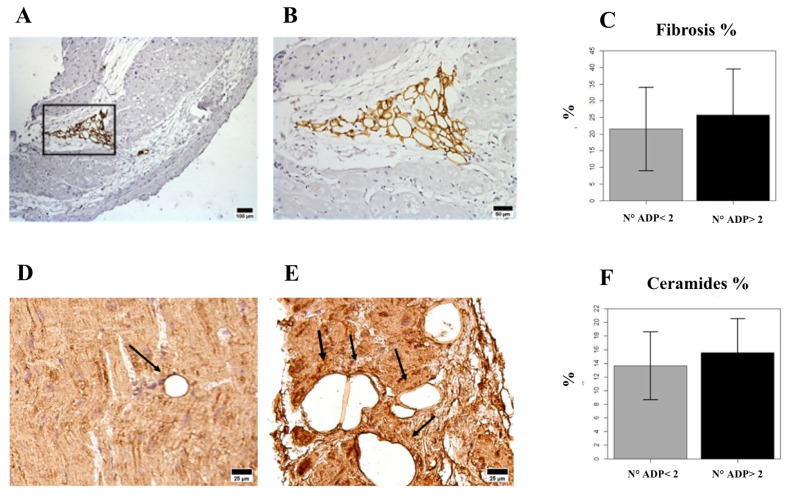
Distribution of PLIN1(+) adipocytes and associated fibrosis and ceramide deposition in LV tissue. (**A**): Interstitial localization of PLIN1(+) adipocytes in the fibrotic regions between cardiomyocytes (scale bar = 100 μm). (**B**): Close-up view of PLIN1(+) adipocytes within fibrotic areas (scale bar = 50 μm). (**C**): Mean percentage ± (SD) of fibrosis in ventricles, categorized according to the presence of low or high levels of PLIN1(+) adipocytes. (**D**,**E**): PLIN1(+) adipocytes with low D or high E ceramide staining (scale bar = 25 μm). (**F**): Mean percentage ± (SD) of the optical density of ceramide staining in ventricles, categorized based on the presence of low or high levels of PLIN1(+) adipocytes. PLIN = perilipin. LV = left ventriculum. SD = standard deviation.

To establish the levels of fibrosis, cell apoptosis, and fat deposition, and the presence of ceramides and myostatin, in the LV samples obtained from patients with AS during surgery, we labeled and quantified these elements using different staining and detection methods, as exemplified in Figure 1. All LV biopsies exhibited positive staining for PLIN5, myostatin, ceramides, fibrosis, and apoptotic cells (Figure 1), but not for PLIN1, which was detected only in a subgroup of samples (n 26). PLIN1 adipocytes tended to be localized close to fibrotic areas as well as in sections of the ventricles with a high positivity of ceramides (Figure 2). Furthermore, after categorizing patients based on the high or low presence of adipocytes in the LV, we found that fibrosis, as well as ceramide levels, were significantly higher in patients with higher detection of PLIN1 adipocytes (Figure 2).

Then, we evaluated the interrelationship between the different histological characteristics of the dysfunctional LV samples obtained from AS patients, after adjustment for BMI (Table 2). We interestingly observed a significant positive association between interstitial fibrosis and the number of apoptotic cardiomyocytes (r = 0.454; *p* = 0.007), as well as between fibrosis and the area covered by PLIN5 (r = 0.389; *p* = 0.019). Apoptosis and PLIN5 also showed a significant association (r = 0.416; *p* = 0.012). Regarding ceramides, we found only a positive trend with fibrosis (r = 0.235) and with PLIN5 (r = 0.241); however, these associations were not statistically significant.

**Figure 3:**
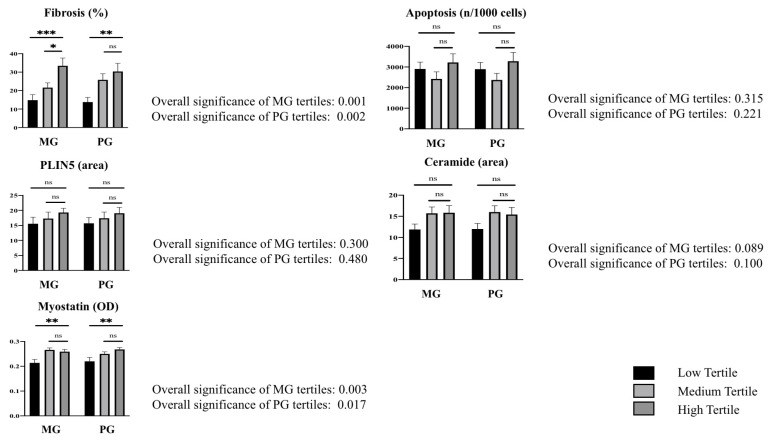
Histological characteristics of the LVs of AS patients, according to the echocardiographic values of mean gradient (MG) and peak gradient (PG). Specific histological features of LVs expressed as means ± SD according to tertiles of MG and PG. The overall significance across groups is also indicated. Significance levels for individual comparisons are identified as * *p* < 0.05, ** *p* < 0.01, and *** *p* < 0.001 for comparison of low and medium tertiles vs. high tertile; ns: no significant difference. LV = left ventriculum. AS = aortic stenosis. SD = standard deviation. PLIN = perilipin.

Afterward, we investigated the correlations between the measured histological features of the LV samples and the values of medium gradient (MG) and peak gradient (PG) obtained from the echocardiography before surgery in patients with AS, dividing the patients into tertiles according to their echocardiographic parameters. As shown in Figure 3, fibrosis in the LV increased significantly with higher levels of MG and PG (both *p* < 0.001). A trend was also observed between PLIN5 and the MG and PG tertiles (Figure 3). Regarding ceramides, they were found in a higher quantity in the tertile with higher MG levels and in a lower quantity in the tertile with lower MG levels (*p* 0.089) (Figure 3). Finally, myostatin was found to be significantly higher in the tertiles with higher MG and PG levels compared with the lower tertiles (*p* 0.003 and *p* 0.017, respectively) (Figure 3).

Subsequently, we performed a multiple linear regression analysis to explore the relationship between fibrosis, which was considered as the dependent variable, and different independent variables, including age, gender, BMI, diabetes mellitus, statin therapy, apoptosis, PLIN5, ceramides, and myostatin. Fibrosis correlated with myostatin (*p* = 0.025; β-coefficient = 0.464), independent from age, BMI, diabetes mellitus, statin therapy, and other histological characteristics of LV, explaining approximately 40% of its variance (R2 0.413) (data not shown in the table). Apoptosis in the model presented an association with fibrosis of borderline significance (*p* = 0.071; β-coefficient = 0.420), independent from the other variables (data not shown in the table).

We also evaluated different functional LV parameters measured with pre-operative echocardiography and their relationships with the measured histological features of the LVs of patients with AS (Table 3).

In particular, we assessed the associations of the E/A ratio (as a marker of LV diastolic function), EF (ejection fraction, as an indicator of heart strength), GLS (global longitudinal strain, as a parameter of systolic function), the DTI septal S’ with the main histological features of the LVs of AS patients, after adjustments for age and BMI (Table 3). We observed a negative association between fibrosis and DTI septal S’ (r = −0.049; *p* = 0.05) as well as a slight negative correlation between fibrosis and GLS (r = −0.266; *p* = 0.086); conversely, there was not any correlation between fibrosis and EF and E/A (Table 3). A borderline positive association was found between apoptosis and E/A (r = 0.270; *p* = 0.082) (Table 3). Additionally, there was a significant positive relation between PLIN5 and E/A (r = 0.444; *p* = 0.009), while there were no correlations between PLIN5 and GLS, EF, and DTI septal S’ (Table 3). Myostatin showed inverse correlations with GLS and EF (r −0.336 and −0.298, respectively) but not with E/A (Table 3). Finally, we did not find any significant associations between histological variables and heart rate, stroke volume, and cardiac output.

## 3. Discussion

In this group of patients undergoing elective surgery for AS, myocardial fibrosis and myocardial steatosis, as evaluated histologically, collectively contributed to ventricular dysfunction. In particular, the degree of fibrosis was correlated with systolic dysfunction. and the degree of fat infiltration in the heart was related to diastolic dysfunction. In this group of patients, an increase in pressure overload, expressed as a mean or peak gradient, was related to increases in LV fibrosis and myostatin and ceramides levels. The levels of ventricular fibrosis were mainly explained by increased myostatin expression, independent from other confounding factors.

AS is a disease that affects both the valve and the myocardium, as myocardial dysfunction is strictly associated with pressure overload [1]. However, the type of ventricular damage in patients with AS has not been well characterized from a histological point of view, nor have these characteristics been correlated with echocardiographic parameters of LV dysfunction.

In this group of patients with severe AS, myocardial fibrosis was strictly related to a reduction in systolic function. Moreover, in the regression model, the amount of fibrosis in LV biopsies was mainly explained by myostatin. Myostatin, a protein of the TGFβ family, was discovered in 1997 in skeletal muscle cells and characterized as a signal that regulates muscle growth and differentiation, as its gene deletion was responsible for the double-muscle phenotype in cattle [11]. In addition, myostatin is known to regulate collagen in the extracellular matrix, and the deletion of myostatin results in fibrosis inhibition [28]. It has been shown that myostatin is also expressed in the hearts of several animals [29,30] and that cardiac fibrosis is decreased in myostatin-deficient mice [31]. In a recent study conducted in myostatin-knockout pigs, the extracellular matrix and total collagen were significantly lower than in wild-type animals [32].

The role of myostatin has also been explored in humans across different types of heart diseases with cardiac remodeling and reduced LV ejection fractions, and a strong association has been found between the severity of the disease and myostatin overexpression [12,13]. Moreover, myostatin levels were increased in adult patients with advanced heart failure in several myopathies as well as in the sera of these patients [12].

In our study in AS patients, myostatin emerged as a significant predictor of ventricular fibrosis, independent of age, gender, BMI, diabetes mellitus, statin therapy, apoptosis, PLIN5, and ceramides, explaining approximately 40% of the variance. In a recent study using a mouse model of AS, treatment with monoclonal anti-myostatin antibodies failed to reduce cardiac hypertrophy or fibrosis [33]. This suggests that myocardial fibrosis and myostatin may not be the only factors responsible for the ventricular dysfunction typical of this disease, thereby highlighting the need to explore other potential research targets.

Myofibrosis and myosteatosis are considered two interrelated mechanisms of muscular damage associated with aging and/or obesity [34,35]. Thus, it would not be surprising to hypothesize that these two phenomena could also be related in the heart.

Cardiac steatosis has been linked with diastolic dysfunction in animal models and has been shown to be an independent predictor of diastolic function in diabetic and obese patients [36]. According to these data on different heart diseases, we also found a strong correlation between diastolic dysfunction and histological markers of myocardial steatosis in AS patients.

Myocardial fibrosis and steatosis may thus contribute to LV insufficiency in AS, mainly affecting global systolic and diastolic functions, respectively. However, several studies using non-invasive imaging have shown that fibrosis could be distributed heterogeneously across ventricular walls in different cardiomyopathies [37,38]. A potential limitation in the design of this study could be the lack of the gold-standard noninvasive method to assess the presence of fibrosis in the entire myocardium, late gadolinium enhancement assessment using CMR [38]. However, it is interesting to note that in our study, not only global markers of whole LV systolic dysfunction but also echocardiographic markers of altered systolic function, measured close to the septal biopsy area, such as DTI septal S’, appeared to be strictly related to the amount of the fibrotic substitution.

In our study, the histological degree of myocardial fibrosis and steatosis were significantly correlated with each other. Moreover, apoptosis resulted in being strictly related not only to myocardial fibrosis but also to myocardial steatosis. From a physiopathological point of view, it is possible to hypothesize that pressure overload causes the death of cardiomyocytes with the consequent release of free fatty acids (FFAs), the recruitment of macrophages, apoptosis, and the activation of profibrotic pathways [10].

In the heart, as in other organs, the excess of free fatty acids starts to accumulate in the form of many different lipid metabolites such as ceramides and other sphingolipids when they cannot be stored in lipid droplets within the cells [39]. Ceramides have recently received great attention as biomarkers of cardiovascular disease and risk [39,40]. From a physiopathological perspective, several studies have demonstrated that ceramides enhance apoptosis, reactive oxygen species (ROS) production, and inflammation [39]. In our study, ceramide levels in LV biopsies were higher in patients within the higher MG and PG tertiles, i.e., with the higher pressure overload. It is interesting to note that ceramides have also been linked to an increased risk of heart failure [21] as they impair the function of cardiomyocytes, damaging their mitochondrial activity and ultimately inducing apoptosis [41]. Ceramides are also emerging as potential inducers of fibrosis, independent of their pro-apoptotic activity, by inducing collagen production [42]. However, the studies on the roles of ceramides in heart tissue and function may be difficult to interpret due to the variations in the specific types of ceramides chosen for the analyses.

Finally, it is known that myocardial steatosis may be due to both lipid droplets stored within cardiomyocytes and triglyceride droplets in cardiac adipocytes [43]. We and others have shown that cardiac adipocytes are present not only as pericardial and epicardial fat but also as intramyocardial adipocytes [18,43]. Interestingly, in the LVs of patients with AS, we found PLIN1 adipocytes close to areas rich in fibrosis or ceramide deposits. Culture studies have shown that cardiac fibroblasts treated with myostatin overexpressed collagen1A- and fibrosis-related pathways [32], supporting a potential direct role of cardiac adipocytes in myocardial damage.

In conclusion, our study provides data on the roles of myocardial fibrosis and steatosis in LV dysfunction, with the unique aspect of direct histological data analysis, which could be, for some aspects, more informative than non-invasive methods. However, further analyses are needed to explore the molecular mechanisms involved in these relationships. It may be important to associate IHC with other complementary methods of analysis, such as, for example, real-time PCR or Western blot, which can assess the expression of target genes or evaluate the proteins involved in the principal molecular pathways. Further studies conducted in wider samples are necessary for better exploring the possible molecular mechanisms and eventual targets of therapies.

Even though our results are only descriptive and require further study, especially to establish the role of cardiac adipocytes, we can speculate that intramyocardial adipocytes may become dysfunctional due to different types of stress. Besides the canonical mechanisms that contribute to adipocytes’ dysfunction, such as inflammation, ROS production, and hypoxia, a role could be also hypothesized for mechanical stress. In a 3D adipocyte culture model, Pellegrinelli et al. [44] showed that mechanical compression of the culture led to decreased lipolysis and an increased expression of pro-fibrotic and pro-inflammatory mediators. The pressure overload from AS could thus alter the transcriptomic expression of different types of LV cells, and, among those, of adipocytes, by stimulating mechanosensitive receptors and pathways.

## 4. Materials and Methods

### 4.1. Population of This Study

We recruited a final group of 31 subjects (19 women and 12 men) undergoing elective cardiac surgery for aortic valve replacement for AS. All patients were affected by severe AS, according to the European Society of Cardiology (ESC) Guidelines of 2012 [45]. Importantly, none of the participants had coronary artery disease deriving from hemodynamically significant stenosis. We included in the analyses only patients treated with ACE inhibitors or sartans, as these drugs are known to play fundamental roles in the cardiac remodeling process induced by wall stress [18]. Moreover, we excluded subjects with weight loss of more than 5% in the previous month, steroid or immunosuppressive therapy in the previous 6 months, renal failure (creatinine values of >2.5 mg/dL), poorly controlled thyroid disease, hormone therapy (except for the thyroid), chronic inflammatory disease, and coronary artery disease. Written informed consent was obtained from each patient, and the study protocol was approved by the Ethics Committee of the University of Verona (protocol number: 381CESC; approval date: 23 December 2014). The characteristics of the study population are shown in Table 1.

### 4.2. Bio-Clinical and Anthropometrical Evaluations

All subjects were clinically evaluated with measurement of their blood pressure and heart rate. Information regarding their smoking habits, the presence of diseases, and medication intake was also collected. Venous blood samples for the metabolic assessments were obtained after overnight fasting. Plasma glucose levels were measured with a glucose analyzer (Beckman Instruments Inc., Palo Alto, CA, USA). Cholesterol and triglyceride levels were determined using a Techincon Auto Analyzer (Techincon Inc., Tarrytown, NY, USA), and dextran–magnesium precipitation was used to derive the HDL values. Weight with an approximation of 0.1 kg (Salus Scale, Milan, Italy) and height using a stadiometer with an approximation of 0.5 cm (Salus Stadiometer, Milan, Italy) were determined for all subjects. Body mass index (BMI) was computed by dividing weight in kilograms by height in square meters. Waist circumference was obtained with a measuring tape at the narrowest circumference of the abdomen.

### 4.3. Echocardiographic Evaluation

All patients underwent a preoperative echocardiogram, performed with an echocardiograph equipped with speckle-tracking analysis (Philips IEE) in the echocardiography clinics at the Cardiology Department of the University Hospital of Verona. For each subject, the following were evaluated: systolic function (the EF of the LV of the biplane Simpson method %), the degree of the AS transvalvular mean gradient (MG), and the peak trans-valve gradient (PG). Furthermore, diastolic function indices were collected using tissue Doppler imaging, such as E/A (the ratio between the E wave, the early diastolic filling component, and the A wave, the atrial diastolic filling component). The pathological cut-off of altered relaxation was defined as E/A < 1 ms. Finally, GLS (LV global longitudinal strain) was measured as an index of systolic functionality, using a two-dimensional speckle-tracking method. The GLS was shown as an absolute value, and we considered normal GLS values of 17.5 ± 3.2%.

### 4.4. Myocardium Collection

In the surgical room, myocardial specimens (interventricular septum tissue) were obtained by trained surgeons after a median sternotomy, approximately 1 h after the induction of anesthesia. The biopsies were then stored for subsequent immunohistochemical evaluation.

### 4.5. Immunohistochemistry (IHC) for Fat Deposition and Myostatin Assay

Freshly isolated ventricular myocardial muscle samples were fixed via immersion in 4% paraformaldehyde in PBS (pH 7.4) overnight at 4 °C, and then dehydrated, cleared, and paraffin-embedded. Five-micrometer-thick serial sections were obtained from the embedded tissues. These sections were then stained with hematoxylin and eosin (H/E) to assess their morphologies prior to immunohistochemical processing. The following primary antibodies were used for the IHC analysis: rabbit anti-human perilipin-1 (PLIN1) (1:150; cat. n°: 9349; Cell Signaling, Danvers, MA, USA) to detect adipocytes, rabbit anti-human perilipin-5 (PLIN5) (1:1000; cat. n°: PA1-46215, Thermo Fisher Scientific, Waltham, MA, USA) to mark lipid droplets inside cardiomyocytes, and rabbit anti-human myostatin (1:25; cat. n°: ab71808, Abcam, Cambridge, UK). The secondary antibody was SignalStain Boost IHC detection reagent HRP Rabbit (ready to use; cat. n°: 8114; Cell Signaling Danvers, MA, USA). A negative control was conducted by using the secondary antibody alone. A diaminobenzidine hydrochloride (DAB) substrate kit for peroxidase was used for the IHC (ImmPACT DAB, cat. n°: SK-4105; Vector Lab, Newark, CA, USA). Briefly, the paraffin-embedded sections were dewaxed and subjected to antigen heat retrieval in sodium citrate buffer (pH 6.00) in a microwave oven for 3 cycles for 5 min each at 750 watt. Endogenous peroxidase activity and non-specific binding were blocked via incubation with 3% hydrogen peroxide (H_2_O_2_) for 10 min and non-immune serum for 60 min at room temperature, respectively. The slides were then incubated sequentially with primary rabbit antibodies for 16 h at 4 °C in a humidified chamber and washed with PBS buffer three times for 5 min each. Subsequently, the sections were incubated with the secondary antibody for 30 min at 20 °C and washed again with PBS for 5 min. DAB was used as the chromogen to visualize peroxidase activity. The sections were counterstained with hematoxylin and were finally assembled with Entellan and overlaid with coverslips.

### 4.6. Immunohistochemistry for Ceramide Assay

The sections were dewaxed and subjected to antigen heat retrieval in sodium citrate buffer at pH 6.00 using a microwave oven for 3 cycles for 5 min each at 750 watts. Endogenous peroxidase activity was blocked by incubating the sections with 3% H_2_O_2_ for 10 min; endogenous biotin and avidin activity were blocked with AvidinBlock and BiotinBlock (Ready to use, Avidin/Biotin Blocking kit, cat. n°: SP-2021; VectorLab, Newark, CA, USA) for 15 min each; and non-specific binding was blocked by incubating the sections with non-immune serum for 20 min at room temperature. The sections were then incubated with primary mouse IgM anti ceramide (1:10; clone MID 15B4, cat. n°: C8104; Sigma-Aldrich, St. Louis, MO, USA) for 16 h at 4 °C in a humidified chamber, washed with PBS buffer three times for 5 min each. Subsequently, the sections were incubated with the secondary antibody Biotinylated Goat anti-mouse IgM (Vectastain ABC Kit Peroxidase HRP, cat. n°: PK-4010; VectorLab, Newark, CA, USA) for 30 min at 20 °C, washed with PBS for 5 min, and incubated with Vectastain ABC reagent (Vectastain ABC Kit Peroxidase HRP, cat. n°: PK-4010; VectorLab) for 30 min at 20 °C. The sections were then washed again with PBS for 5 min. A negative control was conducted by using the secondary antibody alone.

DAB was used as the chromogen to visualize peroxidase activity. The sections were counterstained with hematoxylin, assembled with Entellan, and overlaid with coverslips.

### 4.7. Masson’s Trichrome Staining for Interstitial Fibrosis Assay

Sections of the ventricles were stained with Masson’s trichrome to visualize interstitial fibrosis, according to the manufacturer’s protocol (Trichromic Masson with aniline blue kit, cat. n°: 04-010802; Bio-Optica, Milan, Italy). The sections were deparaffined, rehydrated, stained with ferric hematoxylin according to Weigert for 10 min, incubated with picric acid for 4 min, quickly washed with distilled water, stained with Ponceau B solution for 4 min, quickly washed with distilled water, incubated with phosphomolybdic acid for 10 min, stained with aniline blue according to Masson for 5 min, quickly washed with distilled water, dehydrated, cleared, assembled with Entellan, and overlaid with coverslips.

### 4.8. Apoptosis Assay

The detection of apoptotic cardiomyocytes was performed using the terminal-deoxynucleotidyl-transferase-mediated dUTP nick end labeling (TUNEL) method, according to the manufacturer’s protocol (CardioTACS in SITU Apoptosis Detection Kit, cat. n°: 4827-30-K; Trevigen, Gaithersburg, MD, USA). To apply this method, myocardial tissues from the paraffin-embedded ventricles were cut into thin slices (5 µm thickness). In the TUNEL assay, apoptotic cardiomyocytes were identified by the presence of dark-blue-stained nuclei, while non-apoptotic cardiomyocytes displayed pink/red-stained nuclei.

### 4.9. Image Capturing and Analyses

All ventricle sections were observed at 40×, 200× and 400× magnification using an Olympus BX51 photomicroscope microscope equipped with a KY-F58 CCD camera (JVC) and with Image-ProPlus software (Plus 6.0, NIH, Bethesda, MD, USA) or by using the EVOS FL Auto Cell Imaging System with an EVOS Onstage Incubator (Thermo Fisher Scientific, USA) photomicroscope.

To quantify the number of PLIN1-positive adipocytes among cardiomyocytes, a manual counting method was used. The entire area of each ventricle was observed, and the number of PLIN1-positive adipocytes was manually counted. The total area of the observed section was then calculated using ImageJ software (ImageJ1.8.3) on the acquired images and the number of adipocytes was expressed as cells/mm^2^ by dividing the cell count by the total area expressed in mm^2^.

The optical density (OD) analysis of PLIN5, myostatin, and ceramides was performed on the whole ventricle sections. Images acquired at 200× magnification were analyzed using the Color Threshold function of ImageJ software and manual threshold correction when necessary. All LV biopsies were positive for PLIN5, with a very variable range within the group (mean ± SD: 17.31 ± 6.33 LDs/mm^2^ area; range: 3.21–28.32 LDs/mm^2^ area). Myostatin was detected in all LV samples (mean ± SD: 0.24 ± 0.04 OD; range: 0.14–0.31 OD). All biopsies were also positive for ceramides inside the cardiomyocytes (mean ± SD: 0.24 ± 0.02 OD; range: 0.21–0.31 OD).

ImageJ software was also used to measure the collagen deposition in the ventricle sections. This was achieved by calculating the percentage of blue staining, which serves as an indicator of fibrosis, in relation to the total area of the ventricle section in images acquired at 200× magnification via automated color deconvolution. Manual threshold correction was applied when necessary. With this method, the average percentage of fibrosis was 23 ± 12.94%, with a very variable range (3.11–55.39%) in the study population.

The degree of apoptosis was calculated by counting the number of dark-blue-stained nuclei in cardiomyocytes using ImageJ software on five images acquired at 200× magnification. The degree of apoptosis was then expressed as the number of dark-blue-stained nuclei/mm^2^ and as the average number of cardiomyocytes undergoing apoptosis/10,000 cardiomyocytes [20,22]. The scoring was performed by a single investigator who was blinded to the identities of the samples (mean apoptosis ± SD: 2849.40 ± 1169.23 n/10,000; range: 808.39–5799.96 n/10,000).

### 4.10. Statistical Analysis

The results were reported as means ± standard deviation (SD). Nonparametric tests were used for non-normally distributed variables. Tau-b di Kendall correlations were used to test the level of correlation between the variables. Linear multiple regression was performed to evaluate the effects of the independent variables on the fibrosis-dependent variable. A significance level of 5% was always adopted. All analyses were performed using SPSS (software version 17.0, SPSS).

## 5. Conclusions

In patients with severe aortic stenosis, myocardial fibrosis and myocardial steatosis, which were histologically evaluated, collectively contributed to ventricular dysfunction, correlating with systolic and diastolic dysfunction, respectively. Our study suggests synergistic roles of myofibrosis and myosteatosis in determining ventricular damage in patients with AS and pressure overload. Pressure overload is related to an increase in both myostatin and fibrosis in the myocardium, as well as an increase in myocardial steatosis and ceramide deposition in the ventriculum. Apoptosis is linked to both the degree of fibrosis and steatosis in the LVs of patients with AS.

These associations may highlight potential therapeutic targets to be explored in other experimental models. Furthermore, cardiac adipocytes are also present in the ventricles of patients with AS, close to damaged areas, with a role that is yet to be completely explored.

## Figures and Tables

**Table 1:** The characteristics of the study population (n = 31: 12 male and 19 female subjects).

Variable	M + SD (Range) (n = 31: 12M, 19F)
Age (years)	73.90 ± 5.94 (57–83)
Body mass index (BMI) (Kg/m^2^)	27.37 ± 4.05 (17–70–38.39)
Waist circumference (cm)	96.77 ± 14.42 (55–130)
Systolic blood pressure (mm Hg)	133.64 ± 15.43 (105–175)
Diastolic blood pressure (mm Hg)	69.19 ± 9.09 (55–80)
Total cholesterol (mg/dL)	178.86 ± 40.39 (122–267)
HDL cholesterol (mg/dL)	59.42 ± 17.72 (35–94)
LDL cholesterol (mg/dL)	95.40 ± 37.51(42–196)
Triglycerides (mg/dL)	114.76 ± 45.26 (56–242)
Glucose (mg/dL)	110.58 ± 33.08 (81–217)
Hypertension (n; %)	29 (93.5%)
Diabetes (n; %)	10 (27%)
Statin therapy (n; %)	14 (37.8%)
Oral glucose-lowering drug (n; %)	9 (24.3%)
Insulin therapy (n; %)	3 (8%)
Heart rate (bpm)	71.7 ± 9.8 (52–91)
Stroke volume (mL)	76.9 ± 19.6 (47.2–116.5)
Cardiac output (L/min)	5.3 ± 1.5 (3.3–9)
Medium gradient (MG) (mm Hg)	49.3 ± 12.3 (30–83)
Peak gradient (PG) (mm Hg)	78.3 ± 18.9 (65–98)
E/A	0.86 ± 0.44 (0.42 ± 2.77)
|Global longitudinal strain| (GLS) (%)	14.80 ± 2.5 (9–19)
Ejection fraction (EF%)	59.6 ± 8.4

|GLS| = global longitudinal strain as an absolute value.

**Table 2:** Correlation between the histological characteristics of AS patients’ LV samples after adjustment for BMI.

	Interstitial Fibrosis (%)	Cardiomyocytes Apoptosis (n/10,000)	PLIN 5 (Area)	Ceramides (Area)	Myostatin (OD)
Interstitial Fibrosis (%)	1				
Cardiomyocytes Apoptosis (n/10,000)	0.454 ***p* 0.007**	1			
PLIN 5 (area)	0.389 ***p* 0.019**	0.416 ***p* 0.012**	1		
Ceramides (area)	0.235 NS	0.067 NS	0.241 NS	1	
Myostatin (OD)	0.243 NS	−0.189 NS	0.184 NS	0.035 NS	1

LV = left ventriculum. AS = aortic stenosis. BMI = body mass index. PLIN = perilipin. OD = optical density. NS = no statistical significance.

**Table 3:** Correlations of GLS, EF, E/A, and septum echocardiographic parameters with the main histological characteristics of the LVs of AS patients, after adjustments for age and BMI.

	|GLS|	EF	E/A	DTI Septal S’
**Fibrosis**	−0.266 ***p* 0.086**	−0.148 NS	0.194 NS	−0.409 ***p* 0.05**
**Apoptosis**	−0.148 NS	−0.063 NS	0.270 ***p* 0.082**	0.023 NS
**PLIN5**	−0.177 NS	0.029 NS	0.444 ***p* 0.009**	−0.156 NS
**Ceramides**	−0.126 NS	0.153 NS	0.172 NS	−0.253 NS
**Myostatin**	−0.336 ***p* 0.040**	−0.298 *p* **0.062**	0.067 NS	−0.186 NS

|GLS| = global longitudinal strain as an absolute value. EF = ejection fraction. LV = left ventriculum. AS = aortic stenosis. BMI = body mass index. PLIN = perilipin. DTI septal S’ = tissue Doppler imaging septal S’ wave. NS = no statistical significance.

## Data Availability

The data presented in this study are available upon request from the corresponding author.
